# First record of the male of the widespread *Calliscelio elegans* (Perkins) (Hymenoptera, Platygastridae) along with some taxonomic notes on the species

**DOI:** 10.3897/BDJ.1.e983

**Published:** 2013-09-16

**Authors:** Keloth Rajmohana, Abhilash Peter, TC Narendran

**Affiliations:** † Zoological Survey of India, Calicut, India; ‡ AICOPTAX, Zoological Survey of India, Calicut, India

**Keywords:** *Calliscelio elegans*, male, *Calotelea tanugatra*, new synonymy, Platygastridae, India.

## Abstract

The hitherto unknown male of the widespread and tramp species, *Calliscelio elegans* (Hymenoptera, Platygastridae) is hereby reported for the first time, from India. The two sexes are chromatically quite similar. The male has the same conspicuous banding pattern in the forewing as that of the female. The status of *Calotelea tanugatra* Narendran (Hymenoptera, Platygastridae) from India is reviewed and is proposed to be a junior synonym of *Calliscelio elegans*, new synonymy. Distribution of *Calliscelio elegans* in India is mapped.

## Introduction

*Calliscelio* Ashmead 1893, (Hymenoptera: Platygastridae) with type species *Calliscelio laticinctus* Ashmead is a rather small genus, with 65 species http://hol.osu.edu/index.html?id=461. One strikingly colourful species, *Calliscelio elegans* (Perkins, 1910), is quite widespread throughout the tropics, likely distributed by human commerce, possibly in association with cricket pests (Orthoptera: Gryllidae) of sugar cane, cf. Masner et al. (2009)

**Figure 1.**
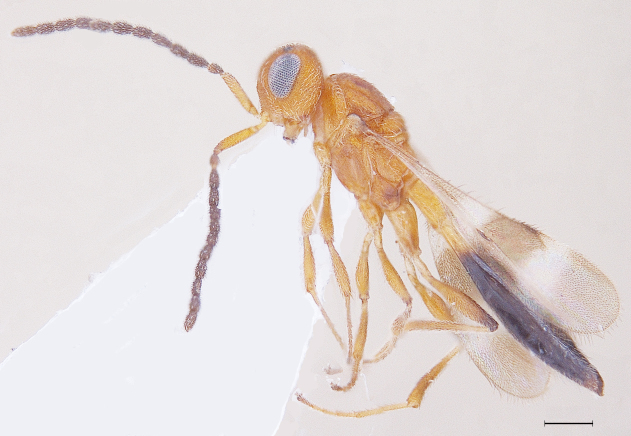
*Calliscelio elegans* Body profile (Male)

During our taxonomic studies on the platygastrid subfamily Scelioninae of India, we identified 12 specimens of *Calliscelio elegans*, from a series of Malaise trap samples from the semi-evergreen forests of Biligirirangan Hills, situated at the conjunction of the Western and the Eastern Ghats in Karnataka, South India. The hitherto unknown male of *Calliscelio elegans* was spotted among the collections, and forms the first ever report of the male of this species. The two sexes are chromatically quite similar (Figs 1, 7). The male has the same conspicuous banding pattern in the forewing (Fig. 8) as that of the female.

We examined the types of Scelioninae at The National Zoological Collection at Western Ghat Regional Centre, Zoological Survey of India and hereby propose *Calotelea tanugatra* Narendran, 1998 (Fig. 9) to be a junior synonym of *Calliscelio elegans* (Perkins).

A distribution map of *Calliscelio elegans* in India is provided (Fig. 12).

## Materials and methods

Specimens for this study belonged to National Zoological Collection, at Zoological Survey Of India, Calicut (ZSIC) and also those received on loan from Western Ghats Insect Inventory Programme of Atree, Bangalore. The description and imaging work were carried out by employing Leica M205A stereomicroscope and Leica DFC-500 digital camera. The species distribution map has been generated using DIVA GIS version 7.4.

Morphological terminology follows (Masner 1980a, Masner 1980c and Mikó et al. 2007). The scale bars in Figs 1, 2, 3, 4, 5, 6, 7, 8, 9, 10, 11 are 0.2mm, except Fig. 7 being 0.5mm.

### Abbreviations

OOL – Ocellocular Length; OD – Ocellar Diameter; POL – Posterior Ocellar Length; IOS – Inter Ocular Space; A1-A12 – antennal segments; T1-T8 – tergites of metasoma; HL – Head Length; HW – Head Width; L – Length; W – Width; ML – Mesosoma Length; MW – Mesosoma Width; MTL – Metasoma Length; MTW – Metasoma Width.

ZSIC – Zoological Survey of India, Calicut.

## Taxon treatments

### Calliscelio elegans

Perkins (1910)

Caloteleia elegans Perkins 1910: 624. Original description.
Caenoteleia elegans Kieffer 1926: 550. Generic transfer, description.
Calliscelio elegans Masner, Johnson & Musetti, 2009: Masner et al. 2009: 61. Description, diagnosis, generic transfer.
Calliscelio elegans *Calotelea tanugatra*Narendran 1998: 71. Female, India (ZSIC) Holotype examined, **syn. nov.**

#### Materials

- **Type status:** Other material. **Occurrence:** recordedBy: Priyadarshan; individualID: ZSI/WGRS/IR.INV.2655; individualCount: 1; sex: Male; lifeStage: Adult; preparations: Card mount; **Taxon:** scientificNameID: Calliscelio elegans; kingdom: Animalia; phylum: Arthropoda; class: Insecta; order: Hymenoptera; family: Platygastridae; genus: Calliscelio; specificEpithet: elegans; scientificNameAuthorship: Perkins; **Location:** continent: Asia; country: India; stateProvince: Karnataka; locality: Biligiriranga Hills; verbatimLocality: Mariappanappala; decimalLatitude: 11.785169 N; decimalLongitude: 77.223671 E; **Identification:** identifiedBy: Rajmohana K; dateIdentified: 2013-6-1; identificationRemarks: First male of the species ever recorded; **Event:** samplingProtocol: Malaise trap; eventDate: 2007-4-20/5-20; habitat: Semi-evergreen; eventRemarks: Collected in Malaise trap; **Record Level:** institutionID: ZSIC
- **Type status:** Other material. **Occurrence:** recordedBy: Priyadarshan; individualID: ZSI/WGRS/IR.INV.2700-2705; individualCount: 5; sex: Female; lifeStage: Adult; preparations: In Alcohol; **Taxon:** scientificNameID: Calliscelio elegans; kingdom: Animalia; phylum: Arthropoda; class: Insecta; order: Hymenoptera; family: Platygastridae; genus: Calliscelio; specificEpithet: elegans; scientificNameAuthorship: Perkins; **Location:** continent: Asia; country: India; stateProvince: Karnataka; locality: Biligiriranga Hills; verbatimLocality: Mariappanappala; decimalLatitude: 11.785169 N; decimalLongitude: 77.223671 E; **Identification:** identifiedBy: Rajmohana K; dateIdentified: 2013-6-1; **Event:** eventDate: 2007-4-20/5-20; habitat: Semi-evergreen; eventRemarks: Collected in Malaise trap; **Record Level:** institutionID: ZSIC
- **Type status:** Other material. **Occurrence:** recordedBy: Priyadarshan; individualID: Atree /BR/10-16; individualCount: 6; sex: Female; lifeStage: Adult; preparations: In Alcohol; **Taxon:** scientificNameID: Calliscelio elegans; kingdom: Animalia; phylum: Arthropoda; class: Insecta; order: Hymenoptera; family: Platygastridae; genus: Calliscelio; specificEpithet: elegans; scientificNameAuthorship: Perkins; **Location:** continent: Asia; country: India; stateProvince: Karnataka; locality: Biligiriranga Hills; verbatimLocality: Mariappanappala; decimalLatitude: 11.785169 N; decimalLongitude: 77.223671 E; **Identification:** identifiedBy: Rajmohana K; dateIdentified: 2013-6-1; **Event:** eventDate: 2007-11-15/12-15; habitat: Semi-evergreen; eventRemarks: Collected in Malaise trap; **Record Level:** institutionID: ZSIC
- **Type status:** Other material. **Occurrence:** recordedBy: Abhilash Peter; individualID: ZSI/WGRS/IR.INV.2485; individualCount: 1; sex: Female; lifeStage: Adult; preparations: Card mount; **Taxon:** scientificNameID: Calliscelio elegans; kingdom: Animalia; phylum: Arthropoda; class: Insecta; order: Hymenoptera; family: Platygastridae; genus: Calliscelio; specificEpithet: elegans; scientificNameAuthorship: Perkins; **Location:** continent: Asia; country: India; stateProvince: Kerala; locality: Trichur; verbatimLocality: Chimmony Damsite; decimalLatitude: 10.523100 N; decimalLongitude: 76.222221 E; **Identification:** identifiedBy: Rajmohana K; dateIdentified: 2013-3-6; **Event:** verbatimEventDate: 2012-1-5; habitat: Mixed Vegetation; eventRemarks: Collected in Sweep net; **Record Level:** institutionID: ZSIC
- **Type status:** Other material. **Occurrence:** recordedBy: Bijoy. C; individualID: ZSI/WGRS/IR.INV.2486; individualCount: 1; sex: Female; lifeStage: Adult; preparations: Card mount; **Taxon:** scientificNameID: Calliscelio elegans; kingdom: Animalia; phylum: Arthropoda; class: Insecta; order: Hymenoptera; family: Platygastridae; genus: Calliscelio; specificEpithet: elegans; scientificNameAuthorship: Perkins; **Location:** continent: Asia; country: India; stateProvince: Kerala; locality: Calicut; verbatimLocality: Jaferkhan Colony; decimalLatitude: 11.266666 N; decimalLongitude: 75.791001 E; **Identification:** identifiedBy: Rajmohana K; dateIdentified: 2011-5-15; **Event:** verbatimEventDate: 2010-9-8; habitat: Mixed Vegetation; eventRemarks: Collected in Sweep net; **Record Level:** institutionID: ZSIC
- **Type status:** Other material. **Occurrence:** recordedBy: Rajmohana.K; individualID: ZSI/WGRS/IR.INV.2637; individualCount: 1; sex: Female; lifeStage: Adult; preparations: Card mount; **Taxon:** scientificNameID: Calliscelio elegans; kingdom: Animalia; phylum: Arthropoda; class: Insecta; order: Hymenoptera; family: Platygastridae; genus: Calliscelio; specificEpithet: elegans; scientificNameAuthorship: Perkins; **Location:** continent: Asia; country: India; stateProvince: Kerala; locality: Calicut; verbatimLocality: Tiruvannur; decimalLatitude: 11.247655 N; decimalLongitude: 75.767650 E; **Identification:** identifiedBy: Rajmohana K; dateIdentified: 2005-12-10; **Event:** verbatimEventDate: 2005-8-11; habitat: Mixed Vegetation; eventRemarks: Collected in Sweep net; **Record Level:** institutionID: ZSIC

#### Description

**Figure 2.**
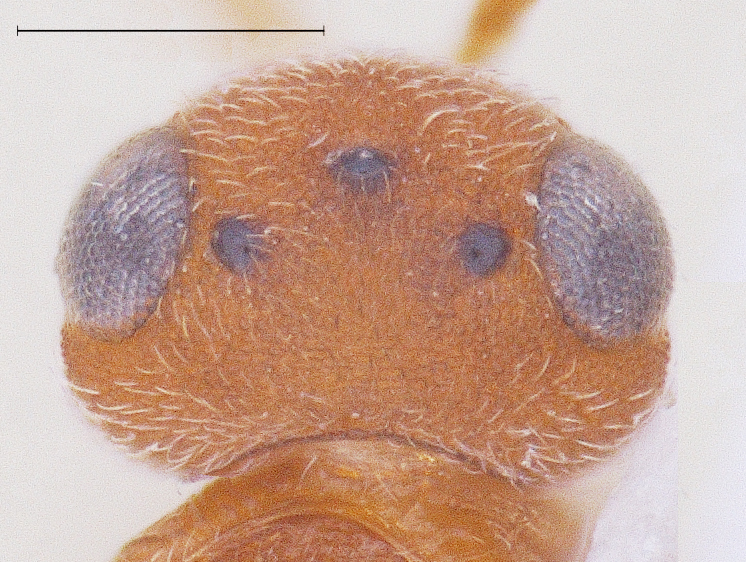
*Calliscelio elegans* Head Dorsal View (Male)

**Figure 3.**
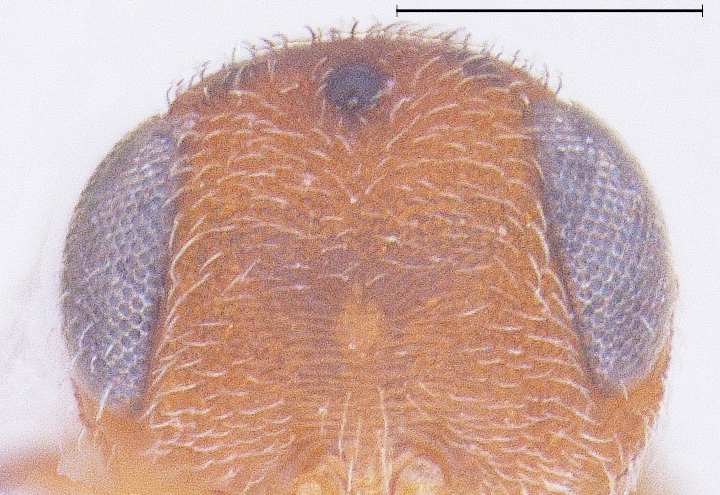
*Calliscelio elegans* Head Front view (Male)

**Figure 4.**
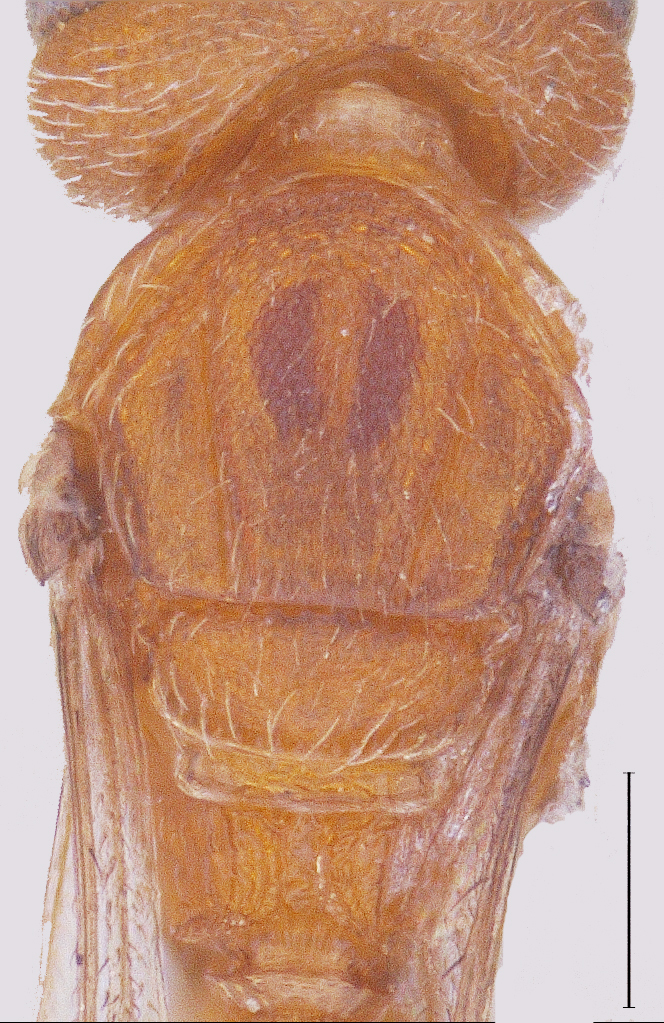
*Calliscelio elegans* Mesosoma dorsal view (Male)

New Description of Male. Length: 1.97mm (n=1) (Fig. 1). Head, mesosoma, T1 wholly and anterior one-fourth of T2, legs and A1 deep orange yellow; posterior three-fourth of T2 onwards till T8, A3-A12 ebony black, A2 and ocelli darkened (Fig. 2); frons (Fig. 3) and mesoscutum medially with a pair of dark patches (Fig. 4); apex of mandibles and apex of hind femur slightly darkened; forewing conspicuously banded, with dark bands basally, medially and apically, separated by light bands (Fig. 8), with median band being most prominent as seen in females (Fig. 7).

Qualitative characters of head and mesosoma being exactly similar to that of female as mentioned in Masner et al. (2009), is not repeated here. However propodeum being flat and elongate, with faint longitudinal striae and irregular rugosities differs from that of the female.

**Figure 5.**
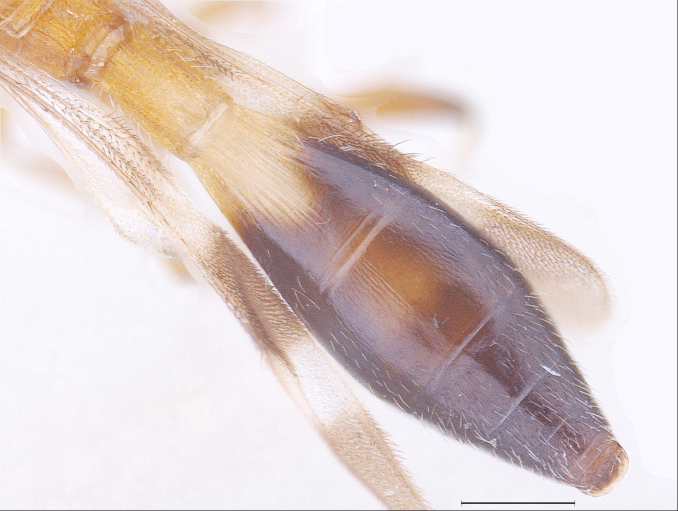
*Calliscelio elegans* Metasoma dorsal view (Male)

Metasoma elongate, widest medially, narrowed both anteriorly and posteriorly (Fig. 5); T1 with fine longitudinal striae and irregular rugose sculpture covering entire surface, almost similar to that on propodeum; longitudinal striae on T2 extending to 0.8 its length medially and about 0.5 of its length laterally. T3 distinctly transverse, shorter than T2, with delicate longitudinal aciculate sculpture, effaced medially; T4-T8 transverse, with delicate coriaceous microsculpture and with abundant appressed golden pilosity.

General body measurements, length to width proportions of antennal segments, forewing and that of metasomal segments are as follows:

Head (dorsal) L:W = 4.05:2.61mm; IOS = 1.14x eye height; POL:LOL:OOL:OD = 1.32:0.75:0.2:0.3.

**Figure 6.**
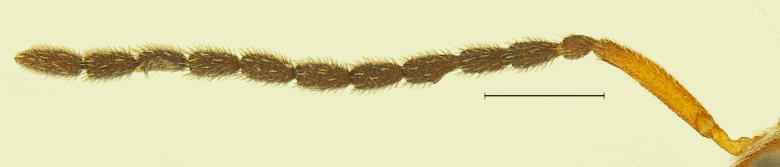
*Calliscelio elegans* Antenna (Male)

A1 4.2x length of radicle, A2 1.05x longer than radicle; A3 and A4 subequal; A5 emarginate and carinate (Fig. 6), 1.15x longer than A4 and 1.02X length of A6; A6 to A8 subequal, A9 to A12 subequal as long as A3, A12 longest, 1.37x A11. Length to width proportions of antennal segments from A1to A12 being 23:5.4, 5.7:4, 8:4, 8:4, 9.2:4.7, 9:4, 9:4, 9:4, 8:4, 8:4, 8:4, 11:4 (Fig. 6).

ML:MW = 4.15: 3.38; Forewing narrow, 5.4x as long as wide, when at rest extending to base of T5; length of veins marginal: stigmal: postmarginal being 6:7:8.

MTL:MTW = 2.03:1.19; T2 longest of all tergites, 1.6x T1 and 1.38x T3 tergite; length to width proportions of T1-T4 being 127:85, 204:170, 148:203, 78:169; T3 onwards transverse; rest of tergites visible as strips; T8 distinct.

#### Diagnosis

##### Variation

Hardly showing any variation from the description of the female by Masner et al. (2009), except for its smaller size (< 2 mm). A pair of dark patches seen on median mesoscutum is not distinct in females. A few of the dense granulations on median frons appear fused as short, coarse irregular strips of striae (Fig. 3), but such a partially striated nature of median frons is seen in all the female specimens of *Calliscelio elegans* included in this study as well.

**Figure 7.**
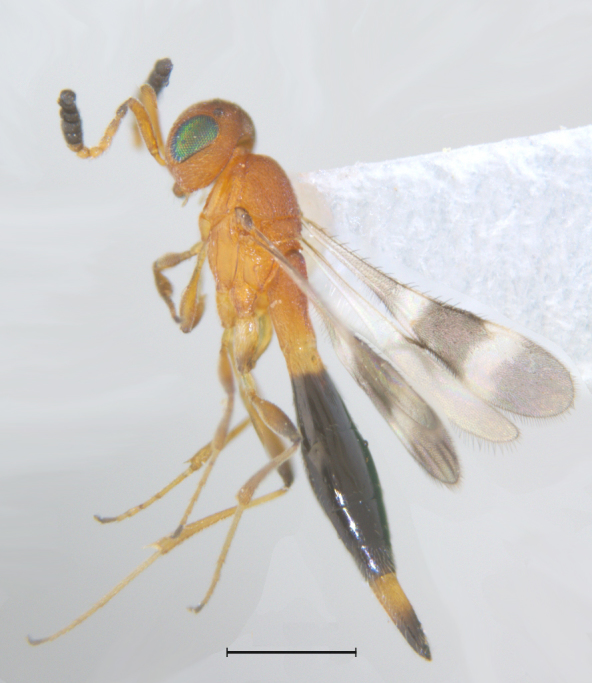
*Calliscelio elegans* Body profile (Female)

**Figure 8.**
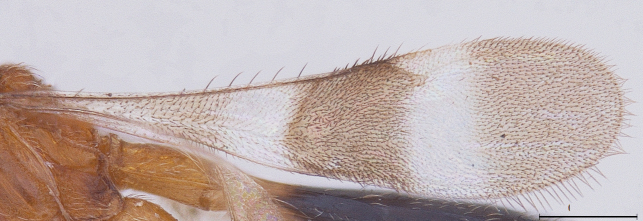
*Calliscelio elegans* Forewing (Male)

The eyes of one of the freshly caught specimen female had a beautiful peacock green metallic lustre (Fig. 7), but turned black within 24 hours of dry preservation.

#### Biology

Egg parasitoid of crickets (Orthoptera: Gryllidae) as per Masner et al. (2009). The females are seen in low numbers, but the males are extremely rare.

#### Ecology

Females are mostly seen close to ground, in search of gryllid eggs for oviposition.

#### Distribution

The species is widely distributed (http://osuc.biosci.ohio-state.edu/hymDB/eol_scelionidae.content_page?page_level=3&page_id=taxon_page_data&page_version=245756&page_option1=M) but generally seen in low numbers (Masner et al. 2009). The distribution of *Calliscelio elegans* in India has been mapped (Fig. 12). It is reported only from the southern states of the country.

### Calotelea tanugatra

Narendran, 1998

**Figure 9.**
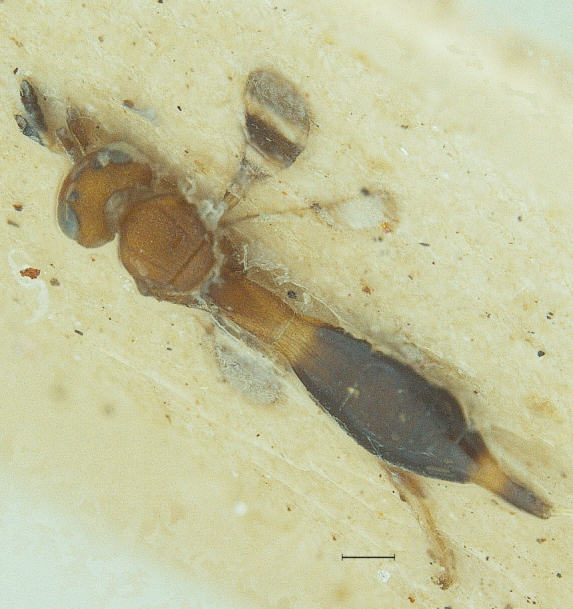
*Calotelea tanugatra* Holotype (Female)

Calotelea tanugatra Narendran (1998), **syn. nov.** (Fig. 9).

#### Materials

- **Type status:** Holotype. **Occurrence:** catalogNumber: ZSIC-1.0189; recordedBy: Narendran TC; individualID: ZSI/WGRS/I.R-INV.1317; individualCount: 1; sex: Female; lifeStage: Adult; preparations: Point Card mount; **Taxon:** kingdom: Animalia; phylum: Arthropoda; class: Insecta; order: Hymenoptera; family: Platygastridae; genus: Calotelea; specificEpithet: tanugatra; taxonRank: species; scientificNameAuthorship: Narendran TC; **Location:** continent: Asia; country: India; stateProvince: Kerala; locality: Malappuram; verbatimLocality: Calicut University Campus; decimalLatitude: 11.169961 N; decimalLongitude: 76.102710 E; **Identification:** identifiedBy: Narendran TC; dateIdentified: 1998; **Event:** year: 1983; month: October; day: 12; habitat: Mixed Vegetation; eventRemarks: Captured by Sweep net; **Record Level:** institutionCode: ZSIC

#### Notes

Quoting Narendran (1998), “forewing with a median blackish brown band surrounded basically and apically by hyaline patches, basal and apical part infumate,”- the banding pattern of forewing (Fig. 9) is correctly described, but the pattern in the illustration as of page 72, does not match the description.

#### Discussion

**Figure 10.**
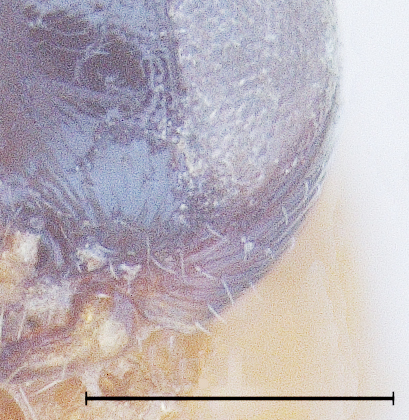
Genal and facial striations of *Calotelea* sp.

**Figure 11.**
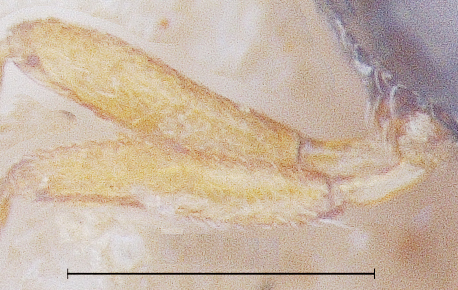
Elongate antennal radicle of *Calotelea* sp.

**Figure 12.**
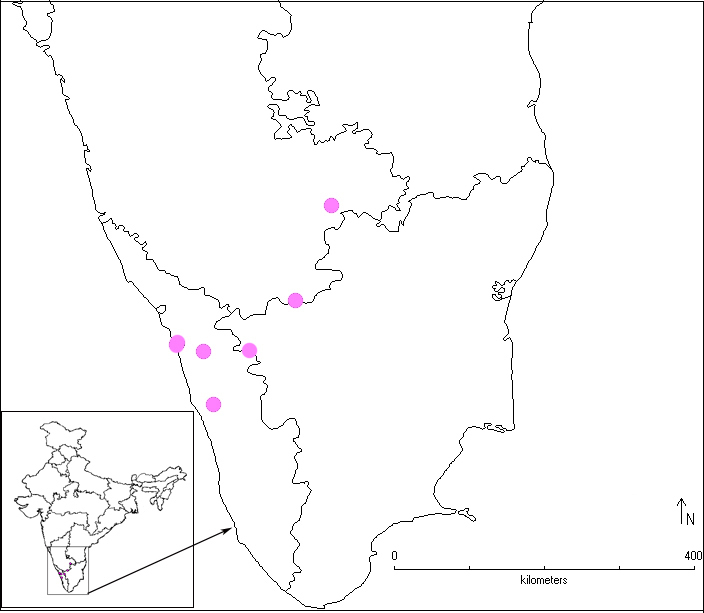
Distribution map of *Calliscelio elegans* in India

As per the generic concept of *Calotelea* Westwood, in Hope (1837), Masner (1976), Masner (1980b), Masner (1980c)​, http://www.zsi.gov.in/right_menu/IIS/index.html and Popovici et al. (2013), diagnosis of the genus from the very similar *Calliscelio* Ashmead, relies on the presence of skaphion in most cases, distinct or at least traces of genal and facial straie (Fig. 10) and an elongate antennal radicle, often measuring about one-third of scape length (Fig. 11). Cheeks and gena are never striate in *Calliscelio* and the antennal radicle is usually short, at most one-fourth length of scape (http://www.zsi.gov.in/right_menu/IIS/index.html). *Calotelea tanugatra* Narendran does not have any traces of striae on mandibular corners or cheeks and antennal radicle is less than one-fourth of scape length. Hence the placement of *Calotelea tanugatra* under *Calotelea* is incorrect. Instead the species agrees in all aspects including the character states, proportions and colouration of the body and wings to *Calliscelio elegans* as stated in Masner et al. (2009). In contrast to most *Calliscelio* species, the metascutellar plate is extremely narrow and weakly concave medially in *Calliscelio elegans*, as seen at times in *Calotelea*. Such a metascutellar plate is seen in *Calotelea tanugatra* too. Hence *Calotelea tanugatra* Narendran (Hymenoptera, Platygastridae) after the current review, is proposed to be a junior synonym of *Calliscelio elegans*, new synonymy.

## Discussion

Masner et al. (2009) studied 60 female specimens of *Calliscelio elegans* from all over the world, but no males were available for their study. From this observed lack of representation of male specimens in the world collections, it was opined that the male sex may either be chromatically different from the female and not so conspicuous or the species can even be thelyotokus. This fact is however disproved by our report and description of the male of *Calliscelio elegans* in this study. It can be concluded that males are extremely rare compared to females and that both the sexes of *Calliscelio elegans* are chromatically quite similar, with the same conspicuous banding pattern on their forewings.

## Supplementary Material

XML Treatment for Calliscelio elegans

XML Treatment for Calotelea tanugatra

## References

[B281213] Ashmead WH (1893). A monograph of the North American Proctotrypidae. Bulletin of the United States National Museum.

[B281244] Hope F. W. (1837). Observations on succinic insects. Transactions of the Entomological Society of London.

[B281254] Kieffer J. J. (1926). Scelionidae. DasTierreich.

[B281264] Masner L. (1976). Revisionary notes and keys to world genera of Scelionidae (Hymenoptera: Proctotrupoidea). Memoirs of the Entomological Society of Canada.

[B281274] Masner L (1980). Key to genera of Scelionidae of the Holarctic region, with descriptions of new genera and species. Memoirs of the Entomological Society of Canada.

[B281284] Masner L. (1980). The identity of Calotelea
ocularis Ashmead, 1894 (Hymenoptera, Proctotrupoidea, Scelionidae). The Canadian Entomologist.

[B281294] Masner L. (1980). A revision of the Nearctic species of Calotelea Westwood (Hymenoptera, Proctotrupoidea, Scelionidae). The Canadian Entomologist.

[B281304] Masner L, Johnson N. F., Musetti L. (2009). Calliscelio
elegans (Perkins), a tramp species, and a review of the status of the genus Caenoteleia Kieffer (Hymenoptera: Platygastridae). Zootaxa.

[B281314] Mikó I., Vilhelmsen L., Johnson N. F., Masner L., Pénzes Z. (2007). Skeletomusculature of Scelionidae. (Hymenoptera: Platygastroidea): head and mesosoma. Zootaxa.

[B281344] Narendran T. C. (1998). A new species and a key to species of Calotelea Westwood (Hymenoptera: Scelionidae) from India. Proceedings of the Zoological Society of Calcutta.

[B281381] Perkins R. C.L. (1910). Supplement to Hymenoptera. Fauna Hawaiiensis.

[B281391] Popovici OA, Masner L, Notton DG, Popovici M (2013). Revision of the European species of Calotelea Westwood (Hymenoptera: Platygastroidea). Zootaxa.

[B281377] Hymenoptera Online (HOL). http://hol.osu.edu/index.html?id=461.

[B281411] Interactive identification system for the common genera of Platygastrid egg parasitoids of rice ecosystem in Kerala (India). http://www.zsi.gov.in/right_menu/IIS/index.html.

[B281483] Platygastroidea. http://osuc.biosci.ohio-state.edu/hymDB/eol_scelionidae.content_page?page_level=3&page_id=taxon_page_data&page_version=245756&page_option1=M.

